# Classification of intestinal inflammation driven by gut microbiota metabolites: a new paradigm for precision treatment of cardiovascular diseases

**DOI:** 10.3389/fmicb.2026.1783688

**Published:** 2026-04-24

**Authors:** Dahua Zhao, Yanjun Cui, Zhen Hua

**Affiliations:** 1 Department of Cardiology, Affiliated Hospital of Shandong University of Traditional Chinese Medicine, Jinan, China; 2 Department of Ultrasound, Affiliated Hospital of Shandong University of Traditional Chinese Medicine, Jinan, China

**Keywords:** cardiovascular disease, gut microbiota, intervention targets, intestinal axis, intestinal inflammation

## Abstract

The global burden of cardiovascular disease continues to increase, and there is an urgent need to revolutionize traditional prevention and treatment strategies. In recent years, in-depth research on the “gut axis” has revealed the core role of gut microbiota and its metabolites in regulating cardiovascular health through intestinal inflammation. However, the heterogeneity of intestinal inflammation limits its clinical treatment and translational efficacy. In this review, we propose a classification system for intestinal inflammation based on the “microbiota metabolite immune disease” axis, which decomposes the general concept of “intestinal inflammation” into five operable molecular subtypes: trimethylamine N-oxide-driven, lipopolysaccharide imbalance, short-chain fatty acid imbalance, aryl hydrocarbon receptor ligand-regulated, and bile acid metabolic disorder types. Each subtype has a unique microbial composition, characteristic metabolite profile, specific receptors, and signaling pathways. Based on this classification, we constructed a personalized and precise treatment approach, with “dietary intervention as the basis, microbial preparations as the core, and drug intervention as the supplement.” In response to the common phenomenon of multiple overlapping subtypes in clinical practice, we propose a personalized adjustment principle of “core contradiction priority, collaborative measures, and taboo avoidance.”

## 1 Introduction

Cardiovascular disease (CVD), an umbrella term encompassing coronary heart disease, myocardial infarction, stroke, heart failure, and hypertension, remains a leading threat to global human health. Given the central role of the gut–cardiac axis in maintaining cardiovascular homeostasis, the development of a classification system grounded in mechanistic insights is increasingly warranted.

The Global Burden of Disease (GBD) study, a large-scale global observational initiative, reports a continued increase in disability-adjusted life years (DALYs) worldwide between 2010 and 2023 (GBD 2023 Diseases and Injuries Collaborators, 2025). CVDs account for a substantial proportion of this burden, with DALYs rising to 437 million (from 320 million in 1990) and global deaths exceeding 19 million in 2023 (GBD 2023 Cardiovascular Diseases and Risk Factors Collaborators, 2025). This upward trend, largely driven by population growth and aging, underscores the need for effective public health strategies and the development of targeted preventive and therapeutic approaches (GBD 2023 Cardiovascular Diseases and Risk Factors Collaborators, 2025).

Faced with the above challenges, the prevention and treatment of CVDs can no longer be limited to traditional cardiovascular risk factors and need to be expanded to cutting-edge interdisciplinary fields. Notably, the gut–cardiac axis, a key mechanism underlying the crosstalk between the gastrointestinal and cardiovascular systems, has emerged as a major focus of interdisciplinary research, providing new insights into CVD pathogenesis and intervention.

Gut microbiota, comprising trillions of microorganisms residing in the human gastrointestinal tract, functions as a complex metabolic organ that contributes to host nutritional metabolism, immune regulation, and barrier integrity through bioactive metabolites such as short-chain fatty acids (SCFAs) and trimethylamine N-oxide (TMAO). High-throughput metagenomic sequencing has identified more than 1,000 bacterial taxa in the human gut, with at least 160 taxa shared across individuals (Qin et al., 2010). Firmicutes and Bacteroidetes represent the dominant phyla; however, the relevance of their relative abundance as a marker of health remains debated (Eckburg et al., 2005). In contrast, Actinobacteria, Proteobacteria, and Verrucomicrobia are present in lower abundance (Gill et al., 2006). Increasing evidence links gut microbiota dysbiosis to the development of CVD (Pinnelli et al., 2025), with TMAO identified as a potential mediator of this association (Wu et al., 2024). Collectively, these findings indicate that gut microbiota and their metabolites influence not only local intestinal homeostasis but also systemic immunity, metabolism, and cardiovascular function through the gut–cardiac axis (Jokar et al., 2025; Dicks, 2025a). Accordingly, further investigation of this axis may provide a valuable framework for understanding CVD pathogenesis and identifying novel preventive and therapeutic strategies.

**Figure 1:**
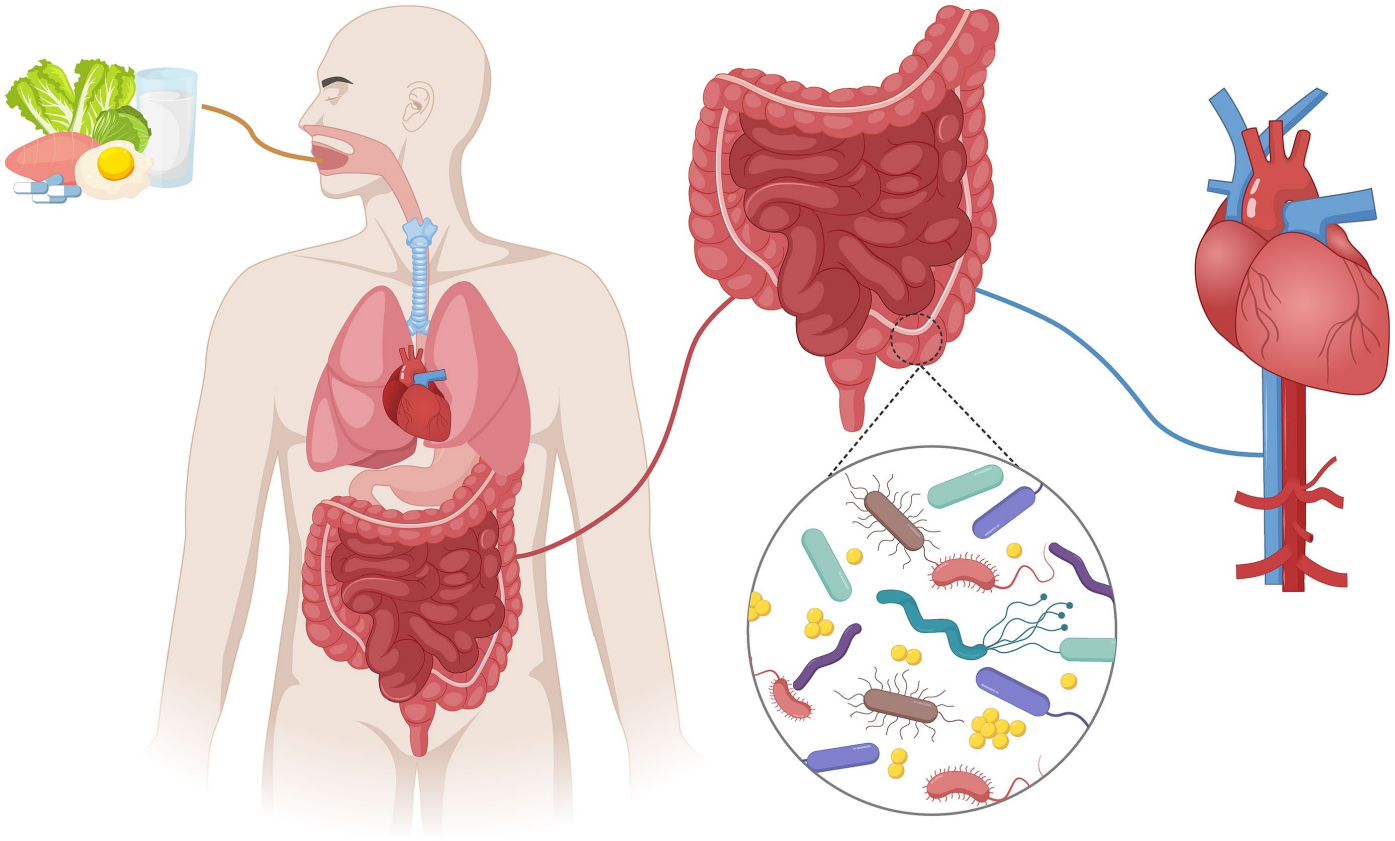
Relationship between gut microbiota and gut axis. The gut microbiota metabolizes food and affects cardiovascular function by regulating host immunity, inflammation, and metabolism.

To achieve targeted prevention strategies, it is crucial to gain a deeper understanding of the mechanisms underlying CVDs. Among them, the discovery of the “gut axis” revealed a bidirectional communication bridge between the gut microbiota and cardiovascular health. Figure 1 depicts the mechanism of gut microbiota involvement in the gut axis. This concept suggests that the gut microbiota interacts with the cardiovascular system through a complex network, in which changes in gut microbiota composition and function can systematically regulate the risk and progression of CVD (Shariff et al., 2024). The core regulatory pathway of the gut axis is gut-derived inflammation driven by microbiota metabolites (dysbiosis of the gut microbiota leads to an abnormal metabolite spectrum, which in turn causes damage to the intestinal barrier, leakage of pro-inflammatory substances, and ultimately leads to chronic inflammation throughout the body). However, there are significant differences in microbiota composition and metabolite spectrum among individuals, resulting in heterogeneous characteristics of gut-derived inflammation. This contradiction has become a key bottleneck for precise prevention and treatment; therefore, a scientific classification system must be established, develop more accurate differential disease diagnosis model. In this review, we propose a classification system for intestinal inflammation based on the “microbiota metabolite immune disease” axis to provide a personalized and precise treatment approach for CVDs.

## 2 Consensus on the mechanisms of intestinal inflammation and heterogeneity challenges

### 2.1 Consensus on the mechanism of intestinal inflammation driving CVD

Animal models and population studies provide key mechanistic evidence for the pathway of gut inflammation caused by abnormal microbial metabolites, which in turn promotes CVD. Research has shown that a diet rich in choline/L-carnitine can enrich specific bacterial genera (such as anaerobic cocci and *Clostridium* in the Firmicutes phylum and *Prevotella* in the Bacteroidetes phylum) and promote TMAO production through the trimethylamine lyase pathway (Shirouchi et al., 2022). TMAO, as the core medium, can promote atherosclerosis by triggering abnormal platelet activation, inhibiting bile acid synthesis, and promoting aortic lesions (Dicks, 2025b). However, in a large, prospective, multi-ethnic/ethnic background cohort, continuous measurement of TMAO over time revealed that there was only a 32% correlation between the high plasma TMAO level and the high risk of atherosclerotic CVD (Budoff et al., 2025), suggesting that TMAO is a moderately strong biomarker and that microbial metabolites are only necessary and insufficient conditions to trigger the intestinal axis. TMAO affects the occurrence and incidence of CVD through inflammation, oxidative stress, endothelial dysfunction, metabolic dysfunction, and other mechanisms (Seldin et al., 2016; Brunt et al., 2020; Gao et al., 2014; Chen et al., 2019; Febbraio et al., 2000; Suzuki et al., 1997; Wang et al., 2011; Zhu et al., 2016). The core mechanism of action of the gut axis has been found to be gut-derived inflammation driven by microbial metabolites. On the one hand, harmful metabolites such as TMAO produced by the metabolism of food by the gut microbiota can disrupt the integrity of the intestinal barrier, leading to “intestinal leakage” and allowing pro-inflammatory substances such as bacterial endotoxins to enter the circulation, causing systemic inflammation (Motte et al., 2025). This chronic inflammation is the key to the development of CVD such as atherosclerosis and heart failure. On the other hand, beneficial metabolites such as butyric acid exhibit significant anti-inflammatory, antioxidant, and metabolic regulatory functions in CVD (Xu et al., 2025).

### 2.2 Heterogeneity challenges of intestinal inflammation

Although scientists have preliminarily revealed the underlying mechanisms, it is difficult to stably replicate the association between changes in microbiota and diseases in large-scale population studies. Therefore, broad-spectrum probiotic therapies designed based on this imprecise association often have limited effectiveness owing to insufficient targeting. This indicates that treating intestinal inflammation as a fixed combination of disease characteristics may be the key to addressing the current research bottleneck. This suggests significant heterogeneity in intestinal inflammation. This heterogeneity is likely due to the unique metabolite lineages generated by different gut microbiota structures, which activate different immune cell subpopulations and downstream signaling pathways by acting on different host receptors, ultimately driving inflammatory responses with different molecular characteristics and leading to diverse cardiovascular pathological phenotypes. Therefore, simply measuring the abundance of a single inflammatory marker or certain microbial communities to achieve effective treatment for different disease courses is challenging. To overcome the current dilemma, it is necessary to go beyond the general understanding of intestinal inflammation and strive for a scientific and systematic classification to transform the vague concept of the “intestinal axis” into a clear pathological framework that can be defined, diagnosed, and accurately intervened.

## 3 Exploration of heterogeneity typing of intestinal inflammatory diseases

**Figure 2:**
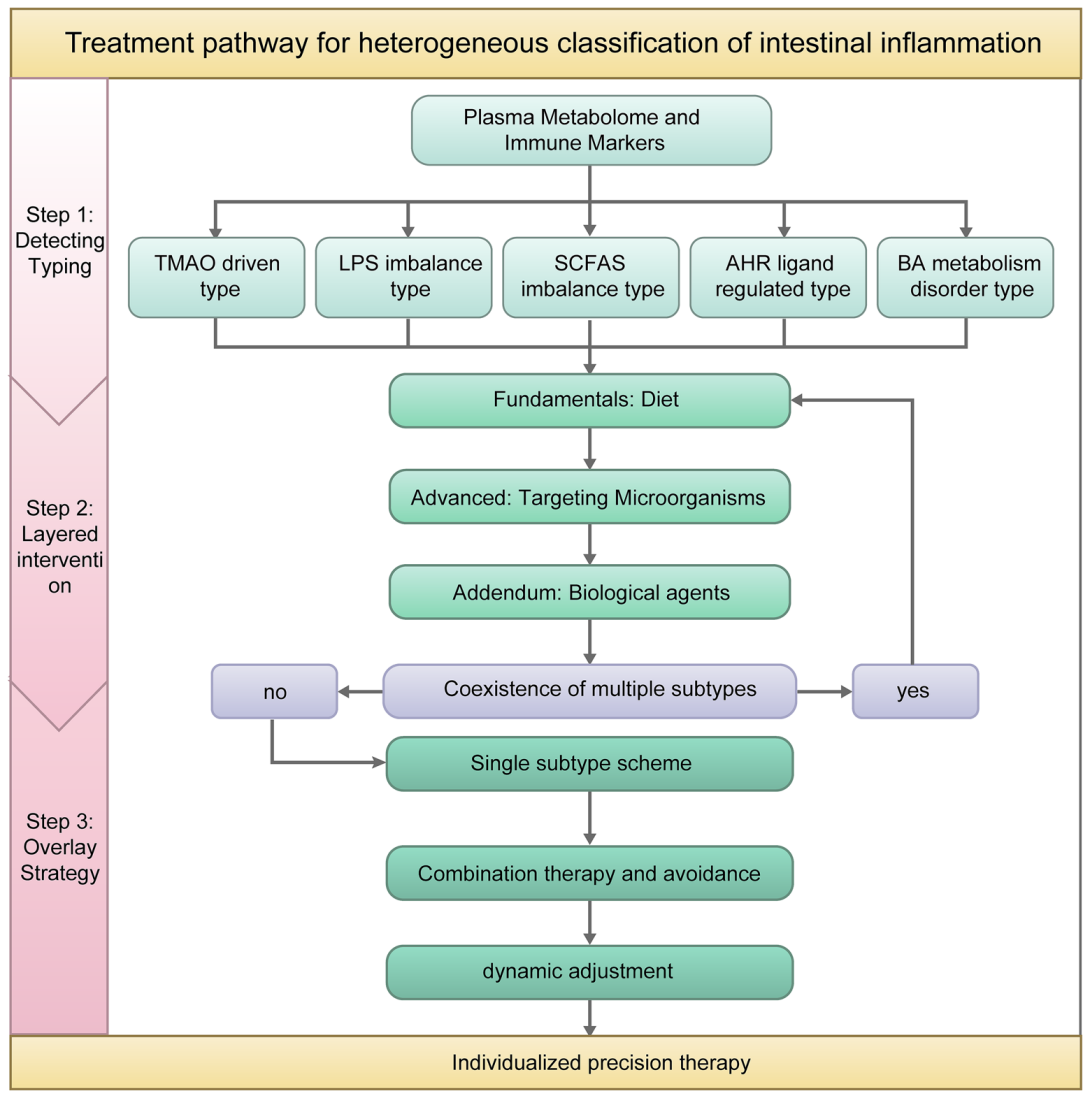
Mechanism diagram of core and multi-subtype crosstalk in five subtypes of intestinal inflammation. This diagram reveals that multiple intestinal metabolites synergistically regulate the expression of inflammatory factors through an interwoven signaling network, with key pro-inflammatory factors such as interleukin (IL)-6, IL-1β, and tumor necrosis factor (TNF)-α serving as common regulatory nodes. Both trimethylamine N-oxide (TMAO) and lipopolysaccharide can aggregate to nuclear factor kappa-light-chain-enhancer of activated B cells (NF-κB) through MAPK/JNK and Toll-like receptor 4 (TLR4) signaling and jointly upregulate the expression of IL-6, IL-1β, and TNF-α. Butyric acid participates in the regulation of these factors through the JNK, ERK1/2, and p38 MAPK pathways, intersecting with the signaling networks of TMAO and lipopolysaccharide. Simultaneously, short-chain fatty acids (SCFAs) regulate T-cell function through the MAPK pathway and affect the levels of IL-17, IL-6, and IFN-γ. Tryptophan metabolites indole-3-propionic acid, indole-3-aldehyde, indole-3-acetic acid promote the expression of anti-inflammatory factors IL-22 and IL-10 induced by regulatory T cells, thereby balancing the aforementioned pro-inflammatory network. Secondary bile acids deoxycholic acid (DCA) and lithocholic acid (LCA) play a bidirectional regulatory role in this network: on the one hand, they inhibit inflammation by activating farnesol X receptor, G protein-coupled bile acid receptor 1, and pregnane X receptor and suppressing NOD-, LRR-, and protein 3 containing the pyrin domain (NLRP3) inflammasome activation; on the other hand, activation of TLR4 and NLRP3 inflammasomes can promote the expression of IL-6, IL-1β, and TNF-α, reflecting their multi-target and context-dependent regulatory characteristics. Metabolic crosstalk is observed in the network: when SCFAs are deficient, the expression of ZO-1, a tight junction protein in the intestinal epithelium, decreases, leading to an increase in lipopolysaccharide translocation and thereby enhancing TLR4/NF-κB signaling; TMAO can inhibit the activity of liver CYP7A1 enzyme, reduce the excretion of primary bile acids, indirectly affect the generation and function of secondary bile acids DCA and LCA, and further connect the regulatory loops between different metabolites.

Based on the above understanding, we classified the heterogeneity of intestinal inflammation. Figure 2 summarizes the characteristics and crosstalk mechanisms of each classification. The human gut microbiota is primarily based on the genetic and functional characteristics of bacteria and is usually divided into five main phyla: Firmicutes, Bacteroidetes, Actinobacteria, Proteobacteria, and Verrucomicrobia (Dauga et al., 2025; Wang et al., 2025; Jain et al., 2025). Although different classification methods may have slight differences, these categories cover >90% of the bacteria in the gut. The above-mentioned gut microbiota can secrete various metabolites, such as TMAO, SCFAs, bile acid, indole-3-propionic acid (IPA), hydrogen sulfide, and phenylacetylglutamine (Hatamnejad et al., 2025). On this basis, this review categorizes intestinal inflammation, with the core classification based on the unique metabolite profiles produced by different microbial communities. These metabolites act on different immune cell subpopulations through specific receptors, driving specific inflammatory responses and CVDs. It can be preliminarily divided into the following categories:

### 3.1 TMAO-driven type

It is a type of intestinal inflammation driven by the gut microbiota that produces trimethylamine lyase. Its core classification feature is the accumulation of the key metabolite TMAO owing to metabolic abnormalities in specific microbiota (Praveenraj et al., 2022). The plasma TMAO levels ≥5 μmol/L can be preliminarily identified as TMAO-driven inflammation (Shanmugham et al., 2023). Dietary components are metabolized in the intestine into precursor compounds (e.g., choline, L-carnitine, and betaine), which are subsequently converted into trimethylamine (TMA) by gut microbiota, primarily members of Firmicutes and Proteobacteria harboring the *cutC* gene (Motte et al., 2025; Wang et al., 2019; Wang and Zhao, 2018; Romano et al., 2015; Amaritei et al., 2025; Craven et al., 2023). TMA is oxidized by flavin monooxygenases in the liver to form TMAO (Saha et al., 2025). TMAO, a key gut microbiota–derived metabolite, contributes to CVD pathogenesis by promoting inflammatory responses and related pathological processes. Mechanistically, TMAO activates NF-κB and NLRP3 inflammasome signaling, induces pro-inflammatory macrophage polarization, and modulates lipid metabolism (Praveenraj et al., 2022; Hayase and Jenq, 2020; Sun et al., 2024; Li et al., 2021). These effects collectively contribute to the development and progression of heart failure, including both ischemic and nonischemic subtypes, through associated signaling pathways and cytokine alterations (Wang Z. et al., 2024; Tang et al., 2014; Salzano et al., 2020; Li et al., 2019). Multiple clinical studies have consistently shown that elevated plasma TMAO levels are significantly positively correlated with the severity and poor prognosis of heart diseases such as myocardial ischemia and heart failure (Tang et al., 2013; Mafune et al., 2016; Yang P. et al., 2024; Avendaño-Ortiz et al., 2023). In summary, TMAO-driven inflammation is classified based on abnormal microbial metabolism (such as members of Proteobacteria and Firmicutes) and demonstrates a clear association between metabolites and cardiovascular inflammation, highlighting the role of intestinal metabolites as contributors to CVD.

### 3.2 Lipopolysaccharide imbalance type

This inflammatory subtype is driven by the translocation of lipopolysaccharide (LPS), a cell wall component of Gram-negative bacteria, into the systemic circulation as a result of impaired intestinal barrier function. In individuals with CVD, LPS levels increase due to intestinal flora imbalance (Tonch-Cerbu et al., 2025). In addition, LPS is a strong risk factor for early arterial atherosclerosis in patients with chronic or repeated bacterial infections (Wiedermann et al., 1999; Vasanthan et al., 2014) and an important driving factor for heart failure with preserved ejection fraction. A plasma LPS concentration >0.5 EU/mL may serve as a preliminary marker for identifying this subtype (Magata et al., 2014). Mechanistically, LPS binds to receptors on immune and endothelial cells, thereby activating inflammatory signaling pathways and inducing the release of pro-inflammatory cytokines (Tonch-Cerbu et al., 2025). This chronic LPS-driven inflammatory response promotes endothelial dysfunction, monocyte recruitment, and plaque progression, thereby accelerating the initiation and development of atherosclerosis (Gorabi et al., 2022; Raetz and Whitfield, 2002). In summary, the LPS imbalance–associated subtype of intestinal inflammation is characterized by elevated circulating LPS levels resulting from impaired intestinal barrier function and microbial dysbiosis. Its significance lies in the well-established association between LPS, an intestinally derived molecule, and CVD development, highlighting the contributory role of gut-derived factors in cardiovascular pathology.

### 3.3 SCFA imbalance type

SCFAs are a class of metabolites produced by the fermentation of dietary fiber by specific symbiotic bacteria in the colon, such as the Firmicutes and Ruminococcus families in the Firmicutes phylum as well as anaerobic bacteria such as *Bifidobacterium*, *Lactobacillus*, *Clostridium perfringens*, and *Vibrio* species. Insufficient intake of dietary fiber can lead to a decrease in the abundance of SCFA-producing bacteria, which in turn reduces the production of their characteristic metabolites such as acetic acid, propionic acid, butyric acid, valeric acid, and isovaleric acid (Tazoe et al., 2008). Elevated levels of acetic acid and decreased levels of butyric acid and propionic acid are considered key metabolic features that distinguish patients with acute myocardial infarction from healthy controls (Avendaño-Ortiz et al., 2023). The typical concentration range of SCFAs in the human colon is 10–20 mmol/kg of butyric acid in feces and 10–30 mmol/kg of propionic acid in feces (Pant et al., 2023). Therefore, butyric acid < 20 mmol/kg and propionic acid <10 mmol/kg in feces can be used as judgment thresholds below the normal reference upper limit to identify SCFA imbalance.

Under normal circumstances, SCFAs have a cardioprotective effect; however, in metabolic imbalance caused by their deficiency, SCFA deficiency contributes to multiple pathological processes, including the induction of inflammatory responses, impairment of cardiac function, and disruption of immune homeostasis (Wang et al., 2023; Yancey, 2005; Liu et al., 2022; Mencarelli et al., 2009; Feng and Xu, 2023; Li et al., 2025c; Li X. et al., 2025). Imbalance of inflammation damages the intestinal mucosal barrier, induces chronic inflammation, and promotes the occurrence and development of CVDs (such as atherosclerosis, hypertension, and heart failure) through the “gut axis” mechanism.

### 3.4 Aromatic hydrocarbon receptor ligand-regulated type

The aryl hydrocarbon receptor (AHR) is a ligand-dependent transcription factor whose ligands primarily include tryptophan-derived metabolites, key intestinal metabolites generated through gut microbial metabolism. As an essential dietary amino acid, approximately 5% of ingested tryptophan is metabolized by gut microbiota, including *Lactobacillus* and *Clostridium* species, via the indole pathway, producing both anti-inflammatory metabolites (e.g., IPA, indole-3-aldehyde [I3A], and indole-3-acetic acid [IAA]) and pro-inflammatory metabolites (e.g., indoxyl sulfate [IS]) (Taleb, 2019; Shah et al., 2025). Tryptophan-derived metabolites that function as AHR ligands contribute to CVD pathogenesis. Under physiological conditions, beneficial AHR ligands, such as I3A, IPA, and IAA, exert anti-inflammatory and tissue-protective effects by modulating immune responses, enhancing intestinal barrier integrity, and maintaining vascular endothelial function (Paeslack et al., 2022; Huang S. S. et al., 2023; Luo et al., 2024; Ganesh et al., 2023; Huang Z. et al., 2023).

In contrast, when metabolic imbalance leads to a decrease in beneficial AHR ligands and the accumulation of harmful metabolites (such as IS), the AHR pathway is abnormally activated, thereby disrupting myocardial cell energy metabolism, inducing cell apoptosis, and promoting the progression of heart failure (Zhang Y. et al., 2025). Elevated IS levels are associated with increased cardiovascular risk, including vascular calcification, myocardial fibrosis, and heart failure (Zhang Y. et al., 2025). Concurrently, reduced levels of beneficial tryptophan-derived metabolites may promote chronic intestinal inflammation, thereby influencing cardiovascular function through systemic immune mechanisms. This indicates that AHR signal imbalance is closely related to the development of vascular inflammation and atherosclerosis (Li et al., 2025b; Wei et al., 2022).

### 3.5 Bile acid metabolism disorder type

Bile acid-regulated inflammation is a type of intestinal inflammation driven by disturbances in bile acid metabolism, mediated by the gut microbiota. A defining feature of this subtype is the microbial conversion of primary to secondary bile acids by gut microbiota, including Bacteroides, Clostridium, and Enterococcus species (Chen W. Y. et al., 2025; Chang et al., 2025b). These gut-derived metabolites contribute to CVD pathogenesis, while under physiological conditions, secondary bile acids exert anti-inflammatory effects through immune modulation (Li et al., 2025a; Zhang J. et al., 2025; Fiorucci et al., 2025; Jia et al., 2025; Kiriyama and Nochi, 2023; Hao et al., 2017). However, when an imbalance in intestinal flora leads to abnormal bile acid metabolism, this anti-inflammatory effect is weakened and even transformed into an inflammatory signal, which in turn drives chronic inflammation by activating inflammatory bodies and increases the risk of atherosclerosis (Chang S. Y. et al., 2025).

In summary, by systematically sorting different types of intestinal inflammation, we gradually elucidated the mechanism of CVDs driven by intestinal inflammation. This review categorizes intestinal inflammation into five types: TMAO-driven, LPS imbalance, SCFA imbalance, aromatic hydrocarbon receptor-regulated, and bile acid metabolism disorder types. Each type has a unique combination of bacteria, metabolic characteristics, signaling pathways, and immune response mechanisms, collectively revealing the complex composition of intestinal inflammation. This classification method not only helps us to have a deeper understanding of how intestinal inflammation occurs but also provides new ideas and basis for prevention and treatment strategies for CVDs.

### 3.6 Mechanism of crosstalk between subtypes of intestinal inflammation

Owing to the extensive metabolic spectrum of the gut microbiota and the interweaving of inflammatory pathways, intestinal inflammation exhibits substantial heterogeneity, contributing to inconsistent findings across microbiome studies in different populations. The proposed classification system addresses this heterogeneity by stratifying patients into distinct subtypes based on plasma metabolomic profiles, immune markers, and gut microbial characteristics, rather than treating intestinal inflammation as a single pathological entity. In individual patients, multiple mechanisms often coexist and interact, forming complex pathological networks. This subtype-based framework enables the identification of dominant pathological drivers and their associated crosstalk, which may underlie variability in research outcomes. By categorizing heterogeneous patients into more homogeneous subgroups with shared core mechanisms, the system reduces confounding arising from population variability and overlapping pathological processes, thereby improving reproducibility and facilitating the development of targeted interventions. When there is crosstalk between SCFA imbalance and LPS translocation and SCFA deficiency occurs, the expression of the intestinal epithelial tight junction protein ZO-1 decreases and intestinal permeability increases (Ferreira et al., 2025). This increases the risk of LPS translocation, forming a vicious cycle of SCFA imbalance causing intestinal leakage and LPS imbalance. When the TMAO-driven type interferes with the bile acid metabolism imbalance type, TMAO can inhibit liver CYP7A1 enzyme activity (a key enzyme for bile acid synthesis) (Ding et al., 2018), reduce primary bile acid excretion, and induce an increase in gut microbiota bile acid hydrolase enzyme activity, leading to secondary bile acid metabolism disorder (Bai et al., 2025). Therefore, in clinical practice, we cannot adopt a “one size fits all” strategy but should follow the diagnosis and treatment approach of holistic regulation and multi-target intervention. By conducting precise individualized assessments of patients, identifying the dominant subtypes of inflammation and potential cross-talk networks in their bodies, and developing targeted comprehensive treatment plans, we can effectively break the vicious cycle of inflammation and achieve improved therapeutic effects.

Based on the unique characteristics of each subtype, we summarize targeted intervention strategies to regulate the key nodes of the subtypes, correct metabolic imbalances, repair immune disorders, improve cardiovascular phenotypes, and achieve corresponding subtype-specific treatment (Table 1).

**Table 1:** Core characteristics of the 5 subtypes of intestinal inflammation driven by gut microbiota metabolites.

No.	Inflammation subtype	Key driver microbiota	Characteristic metabolites	Core receptors/pathways	Major CVD phenotypes	Clinical cutoff
1	TMAO-driven type	Firmicutes, Proteobacteria (cutC+)	TMAO, TMA, choline, L-carnitine	NF-κB, NLRP3 inflammasome	Atherosclerosis, heart failure, thrombosis	Plasma TMAO ≥5 μmol/L
2	LPS imbalance type	Overgrowth of Gram-negative bacteria	LPS, LBP	TLR4, NF-κB	Atherosclerosis, HFpEF	Plasma LPS > 0.5 EU/mL
3	SCFA imbalance type	Decreased SCFA-producing bacteria	Reduced butyrate/propionate; increased acetate	GPR41/43, MAPK	Hypertension, AMI, vascular aging	Fecal butyrate <20 mmol/kg; propionate <10 mmol/kg
4	AHR ligand-regulated type	Lactobacilli, Clostridia (tryptophan metabolism)	Decreased IPA, I3A; increased IS	AHR, Nrf2	Vascular inflammation, heart failure, vascular calcification	Elevated plasma IS; reduced IPA/I3A
5	Bile acid metabolism disorder type	Bacteroides, Clostridium, Enterococcus	Increased primary BAs; decreased secondary BAs	FXR, TGR5, NLRP3	Atherosclerosis, myocardial injury	Abnormal primary/secondary BA ratio

## 4 Treatment pathways and intervention plans based on heterogeneity classification of intestinal inflammation

**Figure 3:**
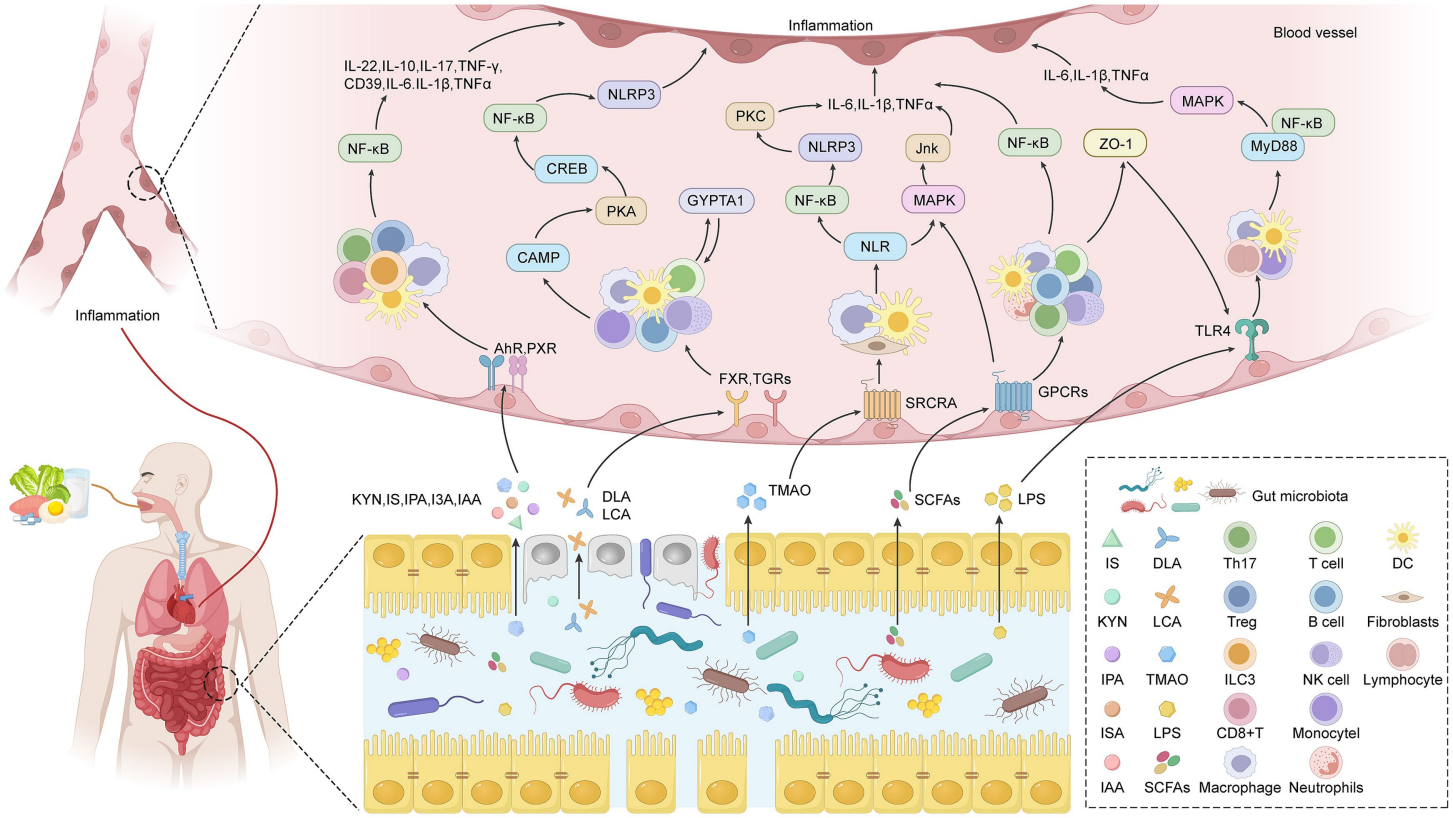
Treatment mind map for patients with various and multiple subtypes. Through multidimensional detection and precise classification, a “diet-based, microbial-based, and drug supplemented” approach is implemented for each subtype. When multiple subtypes overlap, priority-based collaborative intervention is implemented to achieve precise treatment of cardiovascular diseases.

According to the classification, the treatment approach is based on the detection and analysis of the patient’s plasma metabolome and specific immune markers, accurately identifying the predominant subtype of intestinal inflammation. This classification framework addresses heterogeneity in intestinal inflammation and cardiovascular disease research by stratifying patients into distinct mechanistic subgroups. Based on the classification results, targeted intervention strategies can be implemented. Figure 3 shows the treatment mind map for patients with various and multiple subtypes. Table 2 Precision Intervention Strategies for CVD Based on Intestinal Inflammation Subtyping. This classification provides a theoretical basis and potential practical path for promoting individualized prevention and treatment of CVDs.

**Table 2:** Precision intervention strategies for CVD based on intestinal inflammation subtyping.

Inflammation subtype	Dietary intervention (foundation)	Microbiota preparation (core)	Pharmacological intervention (supplement)	Priority in overlapping subtypes
TMAO-driven type	Restrict red meat/dairy ≤50 g/d; replace with soy	*Lactiplantibacillus plantarum* GLP3, Bifidobacterium	Iodomethylcholine, fluoromethylcarnitine, berberine	Highest (acute event-related)
LPS imbalance type	High glutamine (pumpkin, spinach), Mediterranean diet	*Lactobacillus rhamnosus* GG + inulin	Vidarabine, teprenone, statins	Second highest (barrier repair)
SCFA imbalance type	High fiber, polyphenol-rich, low-sodium diet	Bifidobacterium, SCFA-producing probiotics	Oral acetate supplementation	Medium (chronic progression)
AHR ligand-regulated type	Balanced tryptophan, high-quality protein	Indole-producing probiotics	IDO inhibitors, 3-hydroxyaminobenzoic acid	Medium
Bile acid disorder type	Mediterranean diet, high fiber diet	Probiotics, FMT	Obeticholic acid, INT-777, BAR501	Medium

The premise of precise treatment is precise classification. Based on the core characteristics of the five subtypes mentioned earlier, combined with the pathological mechanisms of each subtype, the characteristic detection indicators of different subtypes of intestinal inflammation have been preliminarily clarified. Regarding the TMAO-driven type, the core metabolic markers include plasma TMAO and its precursors (trimethylamine, choline, and L-carnitine), which can be quantified using LC–MS in clinical settings, supporting their potential as clinically applicable biomarkers. The auxiliary immune markers include NF-κB pathway activation products, pro-inflammatory factors (IL-6, IL-1β, TNF-β), and macrophage M1 phenotype markers, which serve as exploratory biomarkers. Microbial detection can target changes in the abundance of Firmicutes, Proteobacteria, Bacteroidetes, *Streptococcus*, and *Pseudomonas*. Regarding the LPS imbalance type, primary detection indicators are plasma LPS and LPS-binding protein levels, which can serve as clinically applicable biomarkers for routine clinical assessment. The key immune markers include TLR4 expression level, NF-κB pathway activity, and pro-inflammatory factors (TNF-α, IL-1β, IL-6), which are considered exploratory markers. Intestinal barrier function markers (such as fecal zonulin and plasma LPS) can assist in determining the degree of “intestinal leakage.” In addition, gut microbial composition may serve as an exploratory biomarker in this context. Regarding the SCFA imbalance type, the core metabolic markers are SCFA components in plasma and feces (decreased levels of butyric acid and propionic acid and relatively increased levels of acetic acid), which serve as clinically ready markers quantified by targeted metabolomics. The immune markers focus on Treg/Th17 balance (decreased proportion of Treg cells and IL-10 levels and increased proportion of Th17 cells and IL-17 levels), which are considered exploratory markers. The microbial community detection is based on the abundance of Firmicutes, *Ruminococcus*, and *Bifidobacterium*. Regarding the AHR ligand-regulated type, metabolic markers need to distinguish between protective and harmful derivatives, namely plasma IPA and I3A (protective) and IS and canine uric acid (harmful) levels, all of which are considered exploratory markers as they are not standardized for clinical use. Immune markers include AHR pathway activity and Treg cell differentiation-related factors (IL-22 and transforming growth factor-β), which also belong to the exploratory category. Regarding the bile acid metabolism disorder type, core detection involves bile acid spectrum analysis (primary bile acid increase and secondary bile acids such as deoxycholic acid and lithocholic acid decrease or depict proportion abnormality), with the total bile acid spectrum (primary/secondary bile acid ratio) serving as clinically ready markers. Immune markers include FXR/TGR5 receptor expression level, NLRP3 inflammasome activity, and dendritic cell maturation markers (CD80, CD86), which are considered exploratory markers.

Current detection technologies enable the simultaneous analysis of multidimensional indicators, thereby facilitating the real-world clinical implementation of this classification system. Plasma metabolic markers can be quantified with high sensitivity using liquid chromatography-mass spectrometry, with a minimum quantification limit of 0.01 μmol/L (Czajkowska et al., 2026). The detection of TMAO and its precursors (carnitine, betaine, and choline) only requires 7.5 μL of plasma, and the sample processing speed is fast (up to 240 samples per day). The sample volume is low (e.g., fingertip blood collection), and isotope labeling standards are not required. It is suitable for clinical batch detection (Rox et al., 2021). Immune markers can be assessed using flow cytometry to characterize immune cell subpopulations and enzyme-linked immunosorbent assays to quantify cytokine profiles, both of which are widely available in routine clinical laboratories (Sapski et al., 2020). Microbial community profiling can be performed using targeted 16S rRNA gene sequencing, which reduces detection costs and turnaround time while potentially lowering overall hospitalization-related expenses (Bajaj et al., 2020). Despite these technological advances, comprehensive multidimensional diagnostics remain more resource-intensive and costly than conventional approaches. To improve feasibility in real-world clinical settings, a layered testing strategy may be adopted. In clinical practice, patient presentations are often heterogeneous due to overlapping inflammatory subtypes, rendering universal multidimensional testing impractical and economically burdensome. A hierarchical approach can balance diagnostic accuracy, cost-effectiveness, and clinical practicality. Specifically, core clinical biomarkers can be applied for initial screening to identify predominant inflammatory subtypes, whereas exploratory biomarkers may be selectively used in complex, refractory, or high-risk cases requiring in-depth mechanistic evaluation. This stratified approach minimizes unnecessary comprehensive testing in low-risk patients, reduces overall healthcare costs and laboratory workload, shortens turnaround time, and enhances the feasibility of implementing subtype-based diagnosis across diverse clinical settings.

### 4.1 Intervention plans for each subtype

For the core pathological processes of different subtypes, an individualized plan based on dietary intervention, with microbial preparations as the core and drug intervention as the supplement, needs to be constructed to achieve precise regulation of the “microbiota metabolite immune” axis. Probiotics, prebiotics, and fecal microbiota transplantation are expected to regulate the composition and activity of the gut microbiome, thereby affecting immune responses and reducing cardiovascular risk factors (Ji et al., 2023; Ciernikova et al., 2023; Wang W. et al., 2024).

#### 4.1.1 TMAO-driven type

In the TMAO-driven models, the core intervention is to reduce TMAO generation and accelerate clearance. At the dietary level, it is necessary to strictly limit the intake of foods that can produce TMAO, such as red meat (≤50 g/d), cod, and dairy products, and replace them with soy products rich in plant protein (Florea et al., 2025; Demarquoy, 2024; Lindqvist et al., 2025; Regis et al., 2025). Microbial intervention involves the use of probiotics, such as *Lactobacillus*, *Bifidobacterium*, and *Escherichia,* which inhibit trimethylamine production (Xie et al., 2021; Zhang S. et al., 2025; Qiu et al., 2017). A randomized controlled trial (RCT) showed that supplementation with *Lactobacillus plantarum* GLP3 for 12 weeks reduced plasma TMAO levels from 284 μg/L to 202.5 μg/L, a decrease of approximately 28.7% (Spasova et al., 2024). Non-antibiotic intervention using a mixture of endogenous/inactivated probiotics can inhibit the activity of trimethylamine lyase, significantly reduce plasma TMAO levels in animal models, and not alter the structure of the gut microbiota, with high stability (Giannini et al., 2025). Oral nano engineered probiotics combine probiotics with polymer nanomedicine; PDMF@LGG is a nano-engineered probiotic system that equips probiotic *Lactobacillus rhamnosus* GG with a polydopamine coating and combines it with reactive oxygen species-responsive nanoparticles of fluorescent methyl choline polymer prodrug. It can inhibit the production of trimethylamine in the intestinal microbiota, strengthen the tight junction of the intestinal epithelium, and reduce the entry of trimethylamine into the bloodstream (Din et al., 2019). At the drug level, the use of iodomethylcholine/fluoromethylcholine, fluoromethylcarnitine, and 3,3-dimethyl-1-butanol (a structural analog of choline) can inhibit microbial trimethylamine lyase without disrupting the balance of the microbiota and has excellent pharmacokinetic properties with a long half-life (Helsley et al., 2022; Cini et al., 2025; Wang et al., 2015). In addition, the natural compound berberine has been shown to inhibit the conversion of trimethylamine (Motte et al., 2025). The above strategy has been preliminarily applied in clinical practice, as shown in randomized crossover trials. Long-term intake of red meat increases plasma TMAO levels by more than twice, and after 4 weeks of meat cessation, TMAO levels significantly decrease (Wang et al., 2019). Thirty patients with a history of atherosclerotic CVD were treated with *Lactobacillus plantarum* GLP3, and 12 weeks of double-blind RCT showed that plasma TMAO decreased from 284 μg/L to 202.5 μg/L, with a decrease of 28.7% (Moffatt et al., 1986). Preclinical studies have shown that berberine reduces TMAO levels in models of hypertension and obesity (Wang et al., 2022). One of the 12-week RCTs targeting healthy young adults found that the effect of seven specific strains of probiotic preparations (including bifidobacteria and lactobacilli) on reducing trimethylamine/TMAO was regulated by individual baseline gut microbiota characteristics (Kaczmarczyk et al., 2025). The study of probiotic preparations emphasized that a “one size fits all” probiotic supplementation strategy may have limited effectiveness. Accurate interventions based on individual gut microbiota characteristics are important for improving the effectiveness of microbial therapy in the future.

#### 4.1.2 LPS imbalance type

Imbalance in the gut microbiota can downregulate junctional proteins, leading to increased intestinal permeability and LPS translocation. Interventions such as the Mediterranean diet, probiotics, and prebiotics (fermentable fibers) can improve intestinal barrier dysfunction by upregulating tight junction protein levels, thereby reducing circulating LPS levels (Violi et al., 2023). Based on this consensus, various targeted intervention strategies have been developed. First, it is necessary to increase the intake of foods rich in glutamine (such as pumpkin and spinach). Glutamine significantly upregulates the mRNA and protein expression of ZO-1 and occludin in intestinal epithelial cells (Wu et al., 2025) and reduces intestinal permeability; glutamine supplementation can reduce serum LPS levels by 45–60%, while inhibiting TLR4/FAK/MyD88 pathway activation and reducing the release of pro-inflammatory factors (TNF-α and IL-6) (Zhang et al., 2022). Clinical studies have shown that the combination of *Lactobacillus rhamnosus* GG and inulin significantly reduces the levels of serum LPS, TLR-4, and IL-6 in patients with coronary heart disease (Asarat et al., 2015). Second, the overall strategy for flora transplantation is crucial. Experimental research has shown that transplanting the intestinal flora of aerobic exercise mice to atherosclerosis model mice can increase the abundance of beneficial bacteria (such as *Prevotella*) and the production of SCFAs, thus repairing the intestinal barrier and inhibiting the development of aortic plaque (Men et al., 2025). Furthermore, targeted application of probiotics with specific functions, such as the strain *Limosilactobacillus reuteri* HY7503, can alleviate endothelial dysfunction by increasing nitric oxide production and regulating cell adhesion molecule expression (Jeon et al., 2024). In addition, the indirect regulation of dietary components is effective. Lactoferrin has been shown to enhance intestinal barrier function by improving dysbiosis of the microbiota, increasing levels of SCFAs, and reducing the translocation of LPS into the systemic circulation (Ding et al., 2025). These multilevel strategies collectively provide empirical evidence and transformation pathways for the prevention and treatment of LPS-related CVDs by repairing the intestinal barrier. Drug intervention with the AC5 inhibitor vidarabine significantly improved mouse cardiac dysfunction induced by *Porphyromonas gingivalis* LPS and alleviated myocardial fibrosis and cell apoptosis (Tsunoda et al., 2023). Shouhui Tongbian Capsule improved heart failure and atrial fibrillation in rats through the microbiota–cardiac axis by restoring the ecological balance of key bacterial genera such as *Turicibacter* in animal models (Chen L. et al., 2025). Teprenone inhibits NF-κB nuclear translocation by activating E3 ubiquitin ligase CHIP, thereby blocking LPS-induced cardiac inflammatory responses (Liu et al., 2024). In addition, statins can effectively reduce atherosclerosis and its complications by regulating intestinal flora imbalance or upregulating intestinal adhesion protein levels (Rosenson, 2004).

#### 4.1.3 SCFA imbalance subtype

The primary therapeutic objective for this subtype is to restore SCFA production and function. A polyphenol-rich diet, including fruits, vegetables, grains, seeds, nuts, and oils, has been shown to increase the abundance of SCFA-producing bacteria (Meiners et al., 2025; Stephen et al., 2017). In addition, reduced sodium intake is associated with increased endogenous SCFA concentrations. Moderate sodium reduction has been reported to alter SCFA levels in patients with untreated hypertension, which is accompanied by decreased blood pressure and improved cardiovascular phenotypes (Chen et al., 2020). Accurate quantification of SCFAs in the human gut is essential for elucidating their role in CVD-related subtypes. Emerging evidence indicates that dietary complex carbohydrates are closely associated with total SCFA production, whereas microbial community composition determines the specific SCFA profile (Arnoldini et al., 2025). As summarized by Chen et al., targeting gut microbiota and its derived SCFAs represents a promising precision strategy for managing cardiovascular diseases, particularly through the modulation of inflammation and endothelial function (Chen et al., 2023). These findings support the rationale for SCFA-based subtype classification.

Biological interventions include the administration of formulations containing specific bacterial strains capable of fermenting dietary fiber (e.g., inulin and pectin) to produce acetate, propionate, and butyrate. A 2024 study demonstrated that supplementation with acetate or a high-fiber diet in aged mice reversed approximately 30% of the age-related increase in pulse wave velocity, a marker of arterial stiffness, and restored carotid endothelium-dependent dilation to levels comparable to those in younger controls, while reducing systemic inflammation (Longtine et al., 2024). This indicates that increasing SCFA levels can effectively improve arterial dysfunction during the aging process. A registered clinical trial (NCT05424263) conducted by the University of Colorado is evaluating the effects of oral acetate supplementation (4,000 mg/day for 12 weeks) on arterial function in adults aged >50 years using a randomized, double-blind, placebo-controlled design exploring whether oral acetate supplements improve arterial function by reducing oxidative stress and increasing the bioavailability of nitric oxide. *Bifidobacterium*, a key SCFA-producing genus, has demonstrated promising effects in CVD-related contexts. A 2025 study reported that supplementation with *Bifidobacterium* in patients with unstable angina (630 mg/day, administered twice daily for 4 weeks) modulated gut microbiota composition and significantly reduced plasma trimethylamine N-oxide (TMAO) levels (Zhou et al., 2025). In addition, a 2024 *in vitro* study showed that *Bifidobacterium longum* L556 modulated the gut microbiota of patients with coronary heart disease, increased the abundance of beneficial bacteria (e.g., *Lactobacillus* species), enhanced SCFA levels, and exerted anti-inflammatory and lipid-regulatory effects (Yang et al., 2025). Furthermore, a 2024 RCT in men with dyslipidemia demonstrated that 12-week supplementation with a multi-strain synbiotic (probiotic–prebiotic combination) significantly increased fecal SCFA levels and serum IL-10, supporting the role of SCFAs in promoting an anti-inflammatory environment, a key mechanism underlying the mitigation of inflammatory CVD (Salamat et al., 2024).

#### 4.1.4 AHR ligand-regulated type

The core intervention for this subtype is to “enhance beneficial metabolites of tryptophan and eliminate harmful metabolites of tryptophan.” The plasma concentration of the beneficial metabolite I3A in patients with atherosclerosis is lower than that in healthy individuals. In animal models, I3A supplementation can reduce vascular inflammation and oxidative stress by activating the AhR-Nrf2 signaling pathway, thus significantly delaying the progression of atherosclerosis (Lu et al., 2023). In patients with chronic kidney disease and heart failure, the level of IS produced by gut microbiota metabolism is elevated, and its concentration is closely related to cardiovascular events and mortality risk (Zhao and Chen, 2022). In atherosclerosis, the upregulation of indoleamine 2,3-deoxygenase stimulates the production of kynurenine, mediates the intensification of AhR-induced vascular inflammation, and promotes foam cell formation. In addition, in cardiac remodeling, kynurenine-mediated activation of AhR aggravates pathological left ventricular hypertrophy and fibrosis (Yang Y. et al., 2024). In response to this association, direct supplementation with beneficial metabolites of tryptophan or probiotics that can produce beneficial indole metabolites (such as specific strains of lactobacilli and bifidobacteria) can promote the function of AHR ligands (Lu et al., 2023). The production of harmful metabolites of tryptophan, such as IS and kynurenine, can be reduced by inhibiting key enzymes (indoleamine 2,3-deoxygenase and tryptophan 2,3-dioxygenase) in their production process (Florance, 1996). 3-hydroxyaminophenylpropionic acid, a downstream metabolite of the kynurenine pathway, is a new drug with anti-inflammatory properties. It can alleviate inflammation and atherosclerosis by regulating 3-hydroxyaminophenylpropionic acid or upstream kynureninase (Melhem and Taleb, 2021). As a downstream metabolite of kynurenine, anthracene acid can alleviate arterial inflammation and reduce cytokine production when orally administered as a derivative of 3,4,-dimethoxycinnamoyl anthracene acid (Cole et al., 2015). In addition, Yixinning Kidney Tablets have been reported to increase serum 5-hydroxytryptamine levels, enhance myocardial energy metabolism, exert anti-inflammatory effects, and improve outcomes in myocardial infarction (Jiang et al., 2022).

#### 4.1.5 Bile acid metabolism disorder type

The core intervention involves regulating the bile acid spectrum and activating the FXR/TGR5 pathway. The basic intervention is to improve the overall intestinal environment and indirectly promote the metabolism of beneficial bile acids by changing dietary habits, consuming high-fiber diets, or switching to a Mediterranean diet (Miyazaki-Anzai et al., 2014). Microbial interventions such as supplementation with specific probiotics and prebiotics (such as chenodeoxycholic acid) and fecal microbiota transplantation can restore gut microbiota balance and increase the production of beneficial secondary bile acids (such as ursodeoxycholic acid and chenodeoxycholic acid), activate FXR/TGR5 receptors, and exert anti-inflammatory effects (Lin et al., 2026; Chang et al., 2025a; Bustamante et al., 2022). Obocholic acid is a small-molecule drug approved by the US Food and Drug Administration. Obocholic acid is a semi-synthetic hydrophobic bile acid analog and a highly selective agonist of FXR (Li et al., 2026; Chapman and Lynch, 2020). Studies have shown that obocholic acid inhibits LPS-induced mitochondrial dysfunction by suppressing the ERK1/2-DRP signaling pathway, protects against LPS-induced myocardial injury, reduces morphological damage to heart tissue, and restores abnormal changes in hemodynamic variables and echocardiographic parameters (Miao et al., 2024; Wu et al., 2019). BAR501 and 6-α-ethyl-23(S)-methylcholic acid (INT-777) were used as agonists of TGR5; the activation of TGR5 reduced the thickness of the aorta and the severity of atherosclerosis in apolipoprotein E deficient (ApoE −/−) mice and reduced the number of plaque macrophages (Biagioli et al., 2023). Mice fed with INT-777 (0.025%) for 3 weeks showed protective changes in cardiac cells; improved myocardial responses to physiological, muscular, and hemodynamic stress; and reduced atherosclerosis (Eblimit et al., 2018).

In clinical practice, most patients have multiple overlapping subtypes, and the pathological mechanisms of each subtype interfere with one another. Therefore, it is necessary to adjust the plan based on the principle of prioritizing core contradictions and coordinating measures based on a single-subtype intervention.

### 4.2 Individualized adjustment strategy for multi-subtype superposition

This classification framework addresses heterogeneity in intestinal inflammation and cardiovascular disease research by stratifying patients into distinct mechanistic subgroups. Based on precise characterization of the microbiota–metabolite–immune axis described above, it provides clearly defined targets for intervention. However, in clinical practice, patients often present with complex profiles in which features of multiple subtypes coexist, with diagnostic thresholds met for more than one subtype simultaneously. The underlying pathological mechanisms may interact, forming interconnected networks. Therefore, future therapeutic strategies should shift from single-subtype interventions toward individualized, precision-based management that integrates multiple subtypes. For patients presenting with overlapping subtypes, a simplified clinical decision-making algorithm may be applied: Step 1: Rapid screening. Assess core clinical biomarkers, including TMAO, LPS, SCFAs, and total bile acids, to identify predominant subtypes. Step 2: Priority ranking. Prioritize subtypes associated with acute cardiovascular events (e.g., TMAO-driven subtype) over those primarily contributing to chronic injury (e.g., SCFA imbalance subtype). Step 3: Integrated intervention. Implement a common foundational dietary strategy, such as a Mediterranean-style high-fiber diet, applicable across multiple subtypes, and select multi-functional microbial interventions to minimize conflicting effects. Step 4: Dynamic adjustment. Reassess relevant biomarkers every 4–8 weeks and adjust interventions according to changes in subtype predominance. In patients with two or more concurrent subtypes, dynamic subtyping should be performed using blood metabolomic profiling (e.g., TMAO, SCFAs, LPS, IS, and secondary bile acids) in combination with immune marker assessment. Intervention priorities should be determined based on the severity of clinical phenotypes and the interactions among underlying mechanisms. Subtypes associated with acute cardiovascular risk, such as the TMAO-driven subtype, should be prioritized, as elevated TMAO levels are associated with an increased risk of thrombosis. In contrast, subtypes contributing to chronic injury, such as SCFA imbalance, may be addressed subsequently, given their role in the progression of atherosclerosis. Dietary intervention serves as a foundational strategy. A personalized high-fiber dietary pattern, based on the Mediterranean diet, is recommended. This approach provides substrates for SCFA production and supplies polyphenols from fruits, vegetables, and nuts that support microbial regulation. In addition, its relatively low content of red meat and saturated fat is beneficial across multiple subtypes, including TMAO-driven, SCFA imbalance, and bile acid dysregulation–associated inflammation. However, potential interactions between dietary and pharmacological interventions should be carefully considered to avoid conflicting mechanisms. For example, the TMAO-driven subtype requires restriction of red meat intake (≤50 g/day) (Jafari et al., 2025), whereas the LPS imbalance subtype emphasizes increased intake of glutamine-rich foods (e.g., pumpkin and spinach). These strategies are compatible and can be implemented concurrently. In contrast, the SCFA imbalance subtype typically requires a high-fiber diet (e.g., inulin and pectin). When combined with bile acid metabolism disorder–associated subtypes, increased fiber intake may enhance bile acid excretion. In such cases, pharmacological support, such as FXR agonists, may be considered to maintain bile acid homeostasis.

When interventions for different subtypes target distinct microbial mechanisms, multi-functional microbial formulations may be preferred over single-strain preparations. Synbiotic formulations combining probiotics and prebiotics, such as *Lactobacillus rhamnosus* GG with inulin, may enhance intestinal barrier integrity, strengthen tight junctions, and reduce LPS translocation. Similarly, combinations such as *Lactobacillus plantarum* GLP3 and *Bifidobacterium longum* have been reported to inhibit TMA production while promoting SCFA generation, thereby targeting multiple pathogenic pathways simultaneously.

## 5 Conclusion

The traditional broad-spectrum framework for the prevention and treatment of CVD requires refinement. Advances in understanding the gut–cardiac axis have highlighted the central role of gut microbiota and their metabolites in cardiovascular homeostasis. This review systematically proposes a heterogeneity-based classification system for intestinal inflammation grounded in the microbiota–metabolite–immune–disease axis. This framework delineates the distinct microbiological, metabolic, and immune mechanisms underlying five key subtypes, thereby transforming the concept of intestinal inflammation into a more precise and actionable pathological framework.

Based on this system, an individualized intervention strategy is proposed, centered on dietary modulation as the foundation, microbial interventions as the core component, and pharmacological therapy as an adjunct. Emerging clinical evidence suggests that this approach may improve metabolic profiles and cardiovascular outcomes. In addition, a management principle for patients with overlapping subtypes—prioritizing dominant pathological drivers, coordinating interventions, and minimizing potential contraindications—is outlined. Collectively, this framework provides a theoretical basis for advancing CVD management from conventional approaches toward precision medicine.

## 6 Discussion and future perspectives

Despite the considerable potential of the proposed classification system to guide precision-based treatment, several challenges remain in its translation from bench to bedside.

### 6.1 Current limitations

First, clinical standardization remains insufficient. The frequent overlap of multiple subtypes within individual patients complicates the application of the current classification system, and existing diagnostic criteria lack adequate standardization. Substantial variability exists in the threshold values of metabolic biomarkers (e.g., TMAO diagnostic thresholds ranging from 4.95 μmol/L to 10 μmol/L) across different disease contexts, populations, and detection platforms (Jaworska et al., 2024; Fang et al., 2025). The absence of unified standards stratified by ethnicity and age limits both comparability and clinical applicability of subtype classification (Budoff et al., 2025).

Second, long-term efficacy data remain limited. Most studies evaluating probiotic and dietary interventions have relatively short follow-up periods (3–12 months), which are insufficient for chronic conditions such as CVD, where long-term (>5 years) data are required to establish sustained efficacy and safety (Rees et al., 2020; Bergum et al., 2021; Hassen et al., 2023).

Third, clinical accessibility remains constrained. The high cost of metabolomic and microbiota-based testing, together with limited insurance coverage for targeted therapies, restricts widespread clinical implementation (Rees et al., 2020; Bergum et al., 2021; Hassen et al., 2023).

### 6.2 Future directions

To address these challenges, future research should focus on three key areas:

(1) Establishing standardized detection systems: Large-scale, multicenter cohort studies are required to develop standardized biomarker detection frameworks stratified by ethnicity, age, and disease stage. Harmonized diagnostic thresholds for key metabolites should be defined. In addition, the development of low-cost, rapid detection technologies (e.g., nanobiosensor-based platforms) may improve accessibility and facilitate implementation in primary care settings (Budoff et al., 2025; Jaworska et al., 2024; Fang et al., 2025).
(2) Conducting long-term prospective studies: Well-designed prospective studies with extended follow-up (>5 years) are necessary to evaluate the durability and safety of subtype-specific interventions. Future research should prioritize clinically meaningful cardiovascular outcomes over short-term metabolic changes and identify optimal intervention windows for each subtype.
(3) Facilitating clinical translation: Strategies to reduce barriers to clinical adoption are essential. These include scaling production of targeted therapeutics and microbial formulations to lower costs, as well as promoting inclusion within healthcare reimbursement systems. Furthermore, integration of multi-omics data with artificial intelligence–based analytical approaches may enhance the precision and scalability of the classification framework. Finally, targeted training of clinicians, particularly in primary care settings, will be critical for effective implementation.

In conclusion, with continued advances in multi-omics and single-cell sequencing technologies, the intestinal inflammation heterogeneity classification system has the potential to evolve from a conceptual framework into a clinically applicable model. Progress in standardization, long-term validation, and cost-effective diagnostics will be essential to enable precision-based prevention and treatment, ultimately providing a novel framework for global CVD management.
